# Self-Assembly Study of Block Copolypeptoids in Response to pH and Temperature Stimulation

**DOI:** 10.3390/polym16081082

**Published:** 2024-04-12

**Authors:** Di Liu, Kang Yang, Liugen Xu, Xiran Shen, Lei Feng, Yangang Jiang, Amjad Ali, Jianwei Lu, Li Guo

**Affiliations:** School of Materials Science & Engineering, Jiangsu University, Zhenjiang 212013, China

**Keywords:** polypeptoid, self-assembly, micelle, pH- and temperature-sensitive

## Abstract

Polypeptoids with well-designed structures have the ability to self-assemble into nanomaterials, which have wide potential applications. In this study, a series of diblock copolypeptoids were synthesized via ring-opening polymerization followed by click chemistry and exhibited both temperature and pH stimulation responsiveness. Under specific temperature and pH conditions, the responsive blocks in the copolypeptoids became hydrophobic and aggregated to form micelles. The self-assembly process was monitored using the UV-Vis and DLS methods, which suggested the reversible transition of free molecules to micelles and bigger aggregates upon instituting temperature and pH changes. By altering the length and proportion of each block, the copolypeptoids displayed varying self-assembly characteristics, and the transition temperature could be tuned. With good biocompatibility, stability, and no cytotoxicity, the polypeptoids reported in this study are expected to be applied as bionanomaterials in fields including drug delivery, tissue engineering, and intelligent biosensing.

## 1. Introduction

Polymer micelles are a class of promising smart materials that has been a subject of extensive interest for both fundamental research and applications in the past few decades [1,2,3]. Polymer micelles fabricated via the self-assembly of amphiphilic molecules can be used in biomedical applications, such as targeting drug carrier systems, tissue engineering, and sensing operations [4,5,6,7]. For example, cancer immunotherapy has been designed to improve our body’s immune system, which is used to recognize and fight cancer [8]. However, there are a lot of challenges, including regarding safety and effectiveness, in cancer immunotherapy, which results in off-target toxicities and severe side effects in the clinical setting [9]. In order to solve these problems, polymeric micelles are employed to deliver cytokines for cancer treatment. Liu et al. reported a system of PMet-P(cdmPEG2K) micelles, which can response to the acidic extracellular tumor environment and inhibit the growth of tumor cells significantly [10,11]. Wrangle et al. encapsulated IL-2 by using PEG-pGlu block copolymer micelles. It was found that the system extended the retention time and improved utilization efficiency of IL-2 [12]. However, some traditional polymer micelles have poor biocompatibility and difficulty responding to changes in the microenvironment of organisms [13].

Polypeptoids, a class of peptidomimetic polymers based on N-substituted glycine backbones, have been attracting more and more attention due to their unique structures and properties [14,15,16,17]. It has been demonstrated that polypeptoids have good biocompatibility because of their structural similarities with respect to polypeptides [18]. The backbones in polypeptoids lack hydrogen bonds and chiral centers, so polypeptiods have flexible skeletons and the properties of polypeptoids are mainly determined by side chains. Polypeptoids are highly designable, and those with specific properties can be synthesized by tuning side chain structures. In particular, some polypeptoids exhibit responses to external stimulation, such as temperature, light, pH, etc. [19,20,21]. Ling et al. reported a diblock polypeptoid, poly(sarcosine-*ran*-butylglycine)-*block*-PNB (P(Sar-*r*-NBG)-*b*-PNB), with both UV- and thermo-responses. The pyrolysis of nitrobenzyl ester pendant groups under 254 nm UV irradiation results in the conversion of lipophilic PNB blocks to hydrophilic polyiminodiacetic acid blocks. Simultaneously, the P(Sar-*r*-NBG) block exhibits thermos-responsiveness. And due to their amphiphilicity, these diblock and triblock copolypeptoids can self-assemble in water and form micelles with PNB blocks as the core [20]. Raczkowska et al. presented novel temperature-responsive polymeric brushes of poly(N-methacryloyl-l-leucine) (PNML) attached to peroxided glass. The PNML coating representative of this type of polymer brush includes a free carboxyl group that can be partially modified to change its transition temperature. Moreover, PNML brushes show high affinity with proteins and excellent cytocompatibility [22]. Researchers have also made great efforts to understand the self-assembly behavior of polypeptoids [23,24,25,26]. Liu et al. reported a supramolecular architectures fabricated from poly(N-allyl glycine) modified with cysteamine (PNAG-NH_2_) and folic acid (FA) via electrostatic interactions. The PNAG-NH_2_/FA complex exhibits a reversible pH-responsive morphological transformation from vesicles (pH = 7.0) to nanofibers (pH = 5.0). They demonstrated that homopolypeptoids electrostatically interact with FA and induce the formation of vesicle nanostructures and fiber arrays. They further encapsulated the anticancer drug doxorubicin (DOX) in the complex vesicle to obtain a pH-induced drug carrier displaying enhanced therapeutic efficacy via typical FA-folate receptor-mediated endocytosis in vitro [25]. Therefore, polypeptoids with properly designed structures can have stimuli responsiveness and self-assemble into nanomaterials. Polypeptoids can be readily created using different preparation methods. In addition to the solid-phase submonomer synthesis method, which affords short-chain polypeptoids, ring-opening polymerization (ROP) has become an effective way of producing high-molecular-weight polypeptoids with different structures in good yields [27,28]. Micelles constructed from polypeptoids, especially those with stimuli-responsive properties, have great application potential yet are underexplored.

In this study, we designed a series of polypeptoids that can form micelles by responding to temperature and pH changes. The polypeptoid diblock copolymers were synthesized via ROP and subsequent click chemistry steps to introduce carboxylic acid groups on the side chains. When dissolved in water, the prepared copolypeptoids can transform from free molecules into micelles reversibly with the change in temperature and pH. The effects of copolypeptoid chain length and block composition on transition temperature in different-pH environments were also studied. These polypeptoids are nontoxic to cells and have great potential for use as drug delivery carriers in the field of biomedicine.

## 2. Experimental Section

### 2.1. Materials

Hydrochloric acid (38%), ethyl acetate (99.5%), phodphorustrichloride (99.5%), ether, and sodium chloride (99.5%) were purchased from Sinopharm Chemical Reagent Co., Ltd. (Shanghai, China). Benzylamine, 2,2-dimethoxy-2-phenylaceto-phenone (DMPA), glyoxylic acid monohydrate, and di-tert-butyl dicarbonate were purchased from Aladdin reagent. Triethyamine, allylamine hydrochloride, methanol, and methyl bromoacetate were purchased from Macklin Co., Ltd. (Shanghai, China). Thioglycolic acid was purchased from Shanghai Acmec Biochemical Co., Ltd. (Shanghai, China). Dichloromethane (DCM), n-hexane, and tetrahydrofuran (THF) were purchased from Shanghai Acmec Biochemical Co., Ltd., and purified by a purification system before use, as required. Deuterated chloroform and anhydrous ethanol were purchased from Sinopharm Chinese Reagent (Suzhou, China). Mouse fibroblasts (L929) were purchased from MeiSen Cell Biotechnology LTD (Hangzhou, China). Culture medium, trypsin, and fetal bovine serum were bought from Cytiva (Logan, UT, USA).

### 2.2. Synthesis of N-Allyl N-Carboxyanhydride (Al-NCA)

2-propen-1-amine hydrochloride (14.1 g) and triethylamine (44 mL) were mixed with 188 mL of ethyl acetate. A total of 17 mL of methyl bromoacetate was added slowly, and the reaction mixture was stirred at 55 °C for 24 h. The product was then extracted using deionized water and saturated brine, and the ethyl acetate was removed using rotavapor. An aqueous solution of HCl (340 mL, 4.0 M) was added, followed by refluxing at 120 °C overnight. The volatile component of the resulting mixture was evaporated, and the residue was recrystallized in methanol/ethyl ether to afford the product (9.9 g, 96.1% yield).

A total of 9.9 g of the above product was dissolved in 260 mL of deionized water. Then, di-tert-butyl dicarbonate (35.7 g) and triethylamine (45 mL) were added to the solution and stirred at room temperature overnight. The mixture was washed by using hexane (3 × 120 mL) to remove unreacted di-tert-butyl decarbonate, followed by adjusting pH to 2 with 4 M HCl aqueous solution. The solution was extracted using ethyl acetate (3 × 120 mL), and the organic phase was washed with brine and dried with anhydrous MgSO_4_. After filtration, the solvent of filtrate was removed, affording a pale brown solid product (8.8 g, 70.2% yield).

A total of 8.8 g of the above product was dissolved in 195 mL of anhydrous CH_2_Cl_2_ under nitrogen atmosphere in a 500 mL flask. A total of 5.4 mL of PCl_3_ was added dropwise to the reaction solution at 0 °C, and then the reaction mixture was stirred for 2 h. The solvent was removed under vacuum, and the residue was extracted with anhydrous CH_2_Cl_2_. The CH_2_Cl_2_ solution was evaporated, and the resulting solid was purified via recrystallization in anhydrous THF/hexane to afford monomer Al-NCA (3.57 g, 61.1% yield).

### 2.3. Synthesis of N-Ethyl N-Carboxyanhydride (Et-NCA)

2-Oxoacetic acid (22.1 g) and ethylamine (6.8 mL) were mixed with 500 mL of dichloromethane and stirred at room temperature for 24 h. The dichloromethane was then removed using rotavapor. An aqueous solution of HCl (150 mL, 4.0 M) was added, followed by refluxing at 120 °C overnight. The volatile component of resulting mixture was evaporated, and the residue was recrystallized in methanol/ethyl ether to afford the product (12.9 g, 77.1% yield).

A total of 12.9 g of the above product was dissolved in 360 mL of deionized water. Then, di-tert-butyl dicarbonate (29.7 g) and triethylamine (64 mL) were added to the solution and stirred at room temperature overnight. The mixture was washed by using hexane (3 × 80 mL) to remove unreacted di-tert-butyl dicarbonate followed by adjusting pH to 2 with 4 M HCl aqueous solution. The solution was extracted with ethyl acetate (3 × 80 mL), and the organic phase was washed with brine and dried with anhydrous MgSO_4_. After filtration, the solvent of filtrate was removed to afford pale yellow oily product (14.0 g, 75.2% yield).

A total of 14.0 g of the above product was dissolved in 328 mL of anhydrous CH_2_Cl_2_ in a nitrogen atmosphere in a 500 mL flask. A total of 9.0 mL of PCl_3_ was added dropwise to the reaction solution at 0 °C, and then the reaction mixture was stirred for 2 h. The solvent was removed under vacuum, and the residue was extracted with anhydrous CH_2_Cl_2_. The CH_2_Cl_2_ solution was evaporated, and the resulting solid was purified via recrystallization in anhydrous THF/hexane to afford monomer Et-NCA (6.2 g, 70.2% yield).

### 2.4. Representative Procedure for the Synthesis of Poly(N-Allyl Glycine)-b-Poly(N-Ethyl Glycine) (PNAG-b-PNEG)

The Al-NCA (120 mg, 0.85 mmol) was dissolved in 1.1 mL of anhydrous THF, followed by adding a stock solution of benzylamine (106 μL, 204 mM in THF). The solution was stirred at 70 °C in a nitrogen atmosphere until all Al-NCA had been consumed. Then, Et-NCA (160 mg, 1.24 mmol) was added to the reaction solution, and stirring was continued at 70 °C until all Et-NCA had been consumed. The final block copolypeptoid was precipitated in excess hexane and then isolated and dried (187 mg, 66.7% yield).

### 2.5. Synthesis of (PNAG-b-PNEG)-COOH via Click Chemistry Procedure Applied to PNAG-b-PNEG and Mercaptoacetic Acid

Mercaptoacetic acid (978 mg, 10.62 mmol), PNAG-b-PNEG (187 mg, 1.06 mmol) ([SH]/[C=C] = 10), and DMPA (15 mg, 0.06 mmol) were dissolved in 2 mL of DMF. The system was degassed and then irradiated with UV light at room temperature for 2.5 h. After being dialyzed for 3 days and lyophilized, a white solid was obtained (207 mg, 73.6% yield).

### 2.6. Cytotoxicity Experiments

The use of a cytotoxicity test is a crucial strategy for testing the safety of biological materials. This experiment incorporated a low-speed centrifuge (SC-3610), a cell incubator (CCL-170B-8), and a multifunctional microplate detector (Synergy H1). Cell Counting Kit-8 (CCK-8) was used to test the cytotoxicity of polypeptoids. Mouse fibroblasts (L929) were inoculated in 96-well plates at a density of 1~2 × 10^4^ cells/well and then incubated in conventional medium (RPMI-1640 with 10% FBS) for 24 h. Polypeptoids were dissolved in conventional medium to form solutions at concentrations of 0 (negative control), 0.125 mg/mL, 0.25 mg/mL, 0.5 mg/mL, 1.0 mg/mL, and 2.0 mg/mL. The solutions were filtered to remove bacteria through the filter. Cells were incubated in 100 μL of the above medium containing polypeptoids for 24 h. After washing the cells with PBS solution, 100 μL of regular medium and 10 μL of CCK-8 were added to each well and incubated for 1~4 h, and then the optical density (OD) at 450 nm was measured (BioTek microplate reader, Winooski, VT, USA).

(1) $$\sigma =\frac{{OD}_{1}-{OD}_{0}}{{OD}_{2}-{OD}_{0}}\times 100\%$$

OD_1_ is the average OD of the experimental group, OD_2_ is the average OD of the negative control group, and OD_0_ is the average OD of the blank control group. The characterization of cytotoxicity entails determining the relative cell viability of the sample solution. A sample is considered non-cytotoxic if the relative viability of the cells is higher than 75%. In order to obtain more convincing experimental results, each sample should be tested at least three times.

### 2.7. Characterizations

The polymeric structural formulas were confirmed using ^1^H NMR (Bruker, 400 MHz, Billerica, MA, USA) spectroscopy. The peaks were referred to in parts per million (ppm) relative to proton impurities of Chloroform-d (CDCl_3_) and Trifluoroacetic acid-d (TFA-d). The chemical bond compositions of monomers and polymers were characterized by employing an attenuated total reflection-Fourier transform infrared spectrometer (Thermo Fisher, ATR-FTIR, Nicolet 8700, Waltham, MA, USA). For tandem gel permeation chromatography (GPC), we adopted the Waters 1525μ (Worcester County, MA, USA) gel permeation chromatograph developed by Waters Corporation of the United States, which is equipped with 2414 differential refractive light detectors. LiBr/DMF (0.05 M) was used as a flowing solution at a flow rate of 1 mL/min. The column temperature was 50 °C, and that of the detector sample cell was 40 °C. The standard curve for molecular weight analysis was prepared with polystyrene as the standard. Dynamic light scattering was performed using a Zetasizer Nano ZS particle size analyzer (Malvern Panalytical, Malvern, UK). A total of 1.5 mL of an aqueous solution of the sample at the appropriate concentration was filtered through a hydrophilic filter (pore size = 0.45 μm) prior to taking differential scanning calorimetry measurements. The test temperature range was 20–70 °C. Z-average diameter and average size distribution were obtained using three measurements. All the cloud point temperature (T_cp_) were measured by monitoring the transmittance of a 450 nm light beam through a quartz sample cell using a Shimadzu Corporation of Japan UV-2700 spectrometer (Kyoto, Japan). The morphologies of polypeptoid micelles were observed using a transmission electron microscope (TEM, JEM-2100, Tokyo, Japan). Micellar solutions at different temperatures were dropwise cast onto copper grids, air-dried at different temperatures, and then negatively stained with 1 wt% uranyl acetate (UA) aqueous solution to prepare TEM samples.

## 3. Results and Discussion

A series of diblock copolypeptoids with both pH- and temperature-responsiveness were designed. These copolypeptoids include PNAG-COOH and PNEG blocks. The PNEG block is soluble in water and does not have any responsiveness. The solubility of the PNAG-COOH block, however, will vary with changes in pH and temperature [29]; thus, the PNAG-COOH block is the responsive block. The copolyeptoids composed of these two blocks are expected to self-assemble into micelles in response to pH and temperature change.

**Scheme 1:**
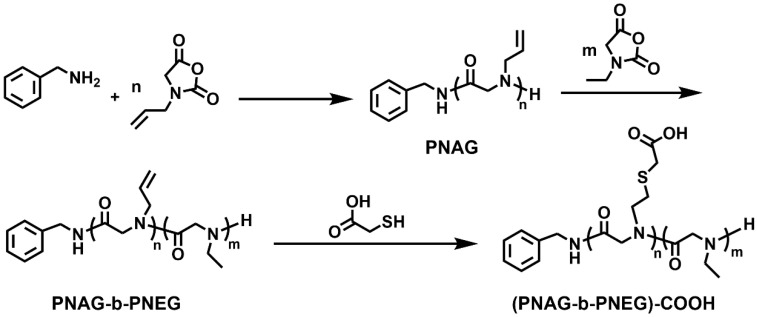
Synthetic pathway of (PNAG-b-PNEG)-COOH.

**Figure 1:**
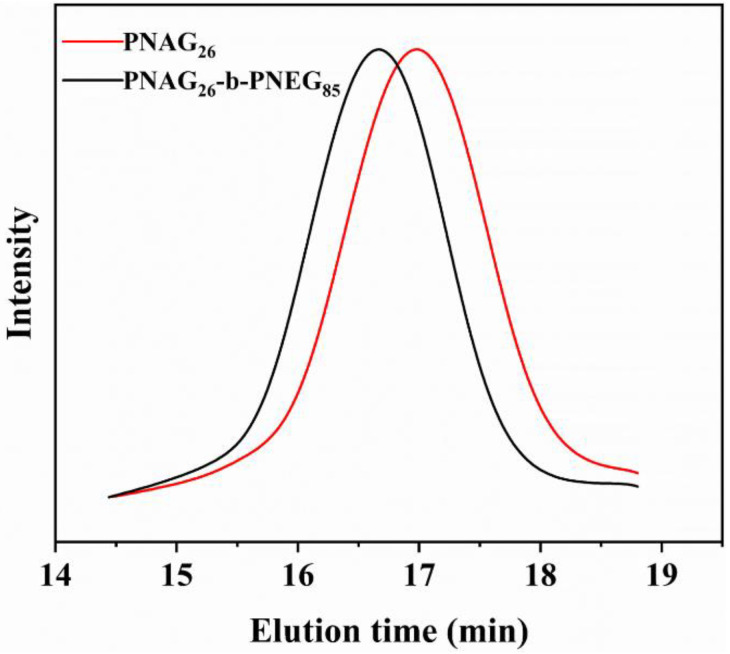
GPC chromatograms of PNAG_26_ and PNAG_26_-b-PNEG_85_.

Two monomers, Al-NCA and Et-NCA, required for polymerizations, were synthesized by optimizing the reported methods (Scheme S1) [30]. The block copolypeptoids PNAG-b-PNEG were synthesized through the ROP of Al-NCA and Et-NCA sequentially added with benzylamine as a nucleophilic initiator (Scheme 1). The progress of polymerization was tracked using FTIR. The disappearance of these monomers’ two characteristic absorption peaks (1770 and 1850 cm^−1^, ν_C=O_) indicated the full transformation of the monomers into polypeptoids (Figure S2) [31]. ^1^H NMR spectra were employed to verify the polymer structures and afford degrees of polymerization (DPs) as well as the compositions of the blocks (Figure S3). Due to the living polymerization nature of this ROP, different compositions of block copolypeptoids were created by adjusting the ratios of initial monomer to initiator and Al-NCA to Et-NCA. All PNAG-b-PNEG samples prepared are summarized in Table 1. The DP_S_ were determined via the proton integral ratios of the allyl or ethyl group on side chains to the phenyl group at chain ends (Figure S3b). The experimental DPs deviated from the theoretical values mainly because of the presence of impurities in the monomers. GPC results demonstrated that the obtained copolypeptoids were monomodally distributed with a relatively narrow molecular weight distribution (polydispersity index, PDI < 1.3). The full shift of the chromatogram peak of PNAG to that of PNAG-b-PNEG suggested that all the PNAG polymer chains were extended with PNEG and block copolypeptoid PNAG-b-PNEG being formed (Figure 1).

**Figure 2:**
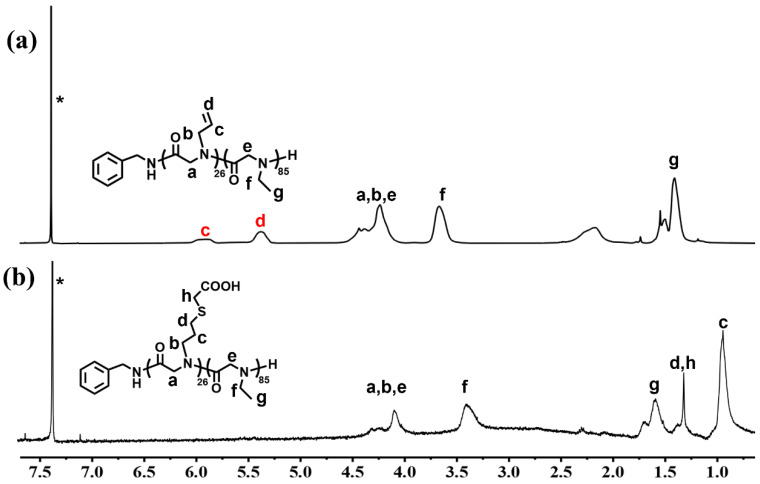
^1^H NMR spectra of (**a**) PNAG_26_-b-PNEG_85_ and (**b**) (PNAG_26_-b-PNEG_85_)-COOH in CDCl_3_ (* indicates CDCl_3_).

**Figure 3:**
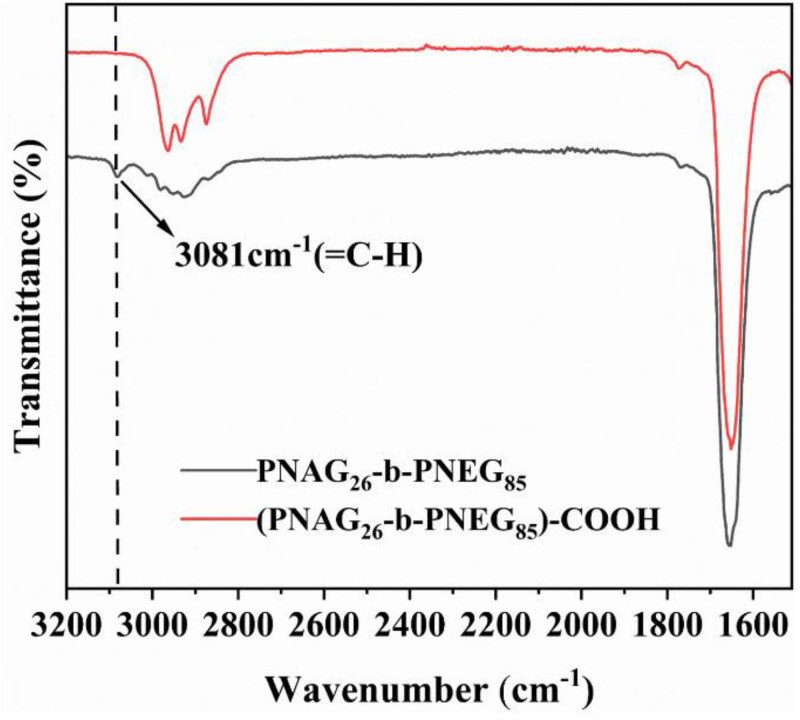
FTIR spectra of PNAG_26_-b-PNEG_85_ and (PNAG_26_-b-PNEG_85_)-COOH.

The final copolymer, (PNAG-b-PNEG)-COOH, was synthesized through the conjugation of thioglycolic acid with the allyl side chains on PNAG-b-PNEG. The disappearance of –CH=CH_2_ peaks (c and d in Figure 2a) and the formation of –CH_2_ CH_2_SCH_2_– peaks (c, d, and h in Figure 2b) in the ^1^H NMR spectra confirmed the quantitative conversion of allyl groups and the success linkage of –COOH on polypeptoid side chains. FTIR analysis was employed to verify the chemical structure of (PNAG-b-PNEG)-COOH (Figure 3). The absence of a characteristic stretching band at 3081 cm^−1^ (ν_=C-H_) indicated full conversion.

**Figure 4:**
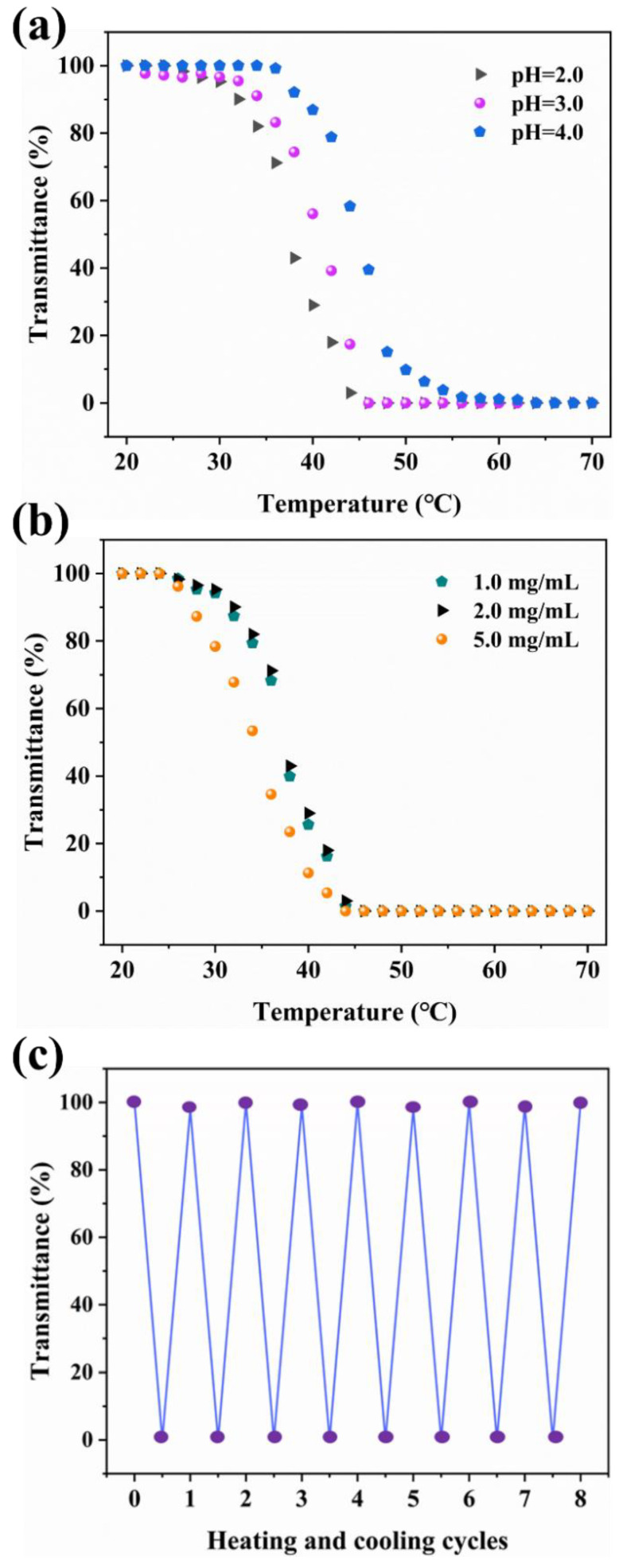
(**a**) Plots of transmittance as a function of temperature for aqueous solutions (2 mg/mL) of (PNAG_26_-b-PNEG_85_)-COOH at different pH levels. (**b**) Plots of transmittance as a function of temperature for aqueous solutions of (PNAG_26_-b-PNEG_85_)-COOH at pH = 2.0 at different concentrations. (**c**) Transmittance of (PNAG_26_-b-PENG_85_)-COOH aqueous solution at a concentration of 2.0 mg/mL at pH = 2.0 (8 heating and cooling cycles between 20 and 70 °C).

The final block copolypeptoids, (PNAG-b-PNEG)-COOH, synthesized via click chemistry were expected to exhibit temperature- and pH-responsive behavior in acidic solutions due to the existence of the responsive part: the PNAG-COOH block. Under different pH conditions, the (PNAG-b-PNEG)-COOH copolypeptoids containing carboxyl groups exhibit different degrees of protonation. In addition, there are interchain and intrachain hydrogen bonds between the amide group on the backbone and the carboxyl group on the side chain. The polypeptoids will form hydrogen bonds with water. The existence of hydrogen bonds also affects their thermal response behavior [29]. The desired pH value was obtained by adjusting HCl solutions, and the (PNAG-b-PNEG)-COOH copolypeptoids were dissolved at different concentrations to afford experimental samples. It was clear that the copolypeptoid solutions became cloudy upon heating. To better describe the transition response to temperature and pH, UV-Vis spectroscopy was employed to characterize the cloud-point temperature (T_cp_). T_cp_ was defined as the temperature at 50% UV-Vis transmittance (λ = 450 nm) of the sample solution. As shown in Figure 4a, the transmittance of the (PNAG_26_-b-PNEG_85_)-COOH solution in an acidic environment (pH = 2.0, 3.0, and 4.0) decreased gradually from 100% until it reached zero following a change in temperature, which suggested the solution would transform from clear to cloudy. The T_cp_ decreased when the pH decreased. The T_cp_ dropped from 45 to 37 °C when the pH value changed from 4.0 to 2.0 (Figure 4a and Table S1). These changes in response to temperature and pH can be attributed to the solubility change of the carboxyl-group-containing chain segment, namely, the PNAG-COOH block in the diblock copolypeptoid. The PNAG-COOH block became insoluble in water when the temperature increased or the pH value decreased, resulting in the aggregation and precipitation of the copolypeptoid. T_cp_ can be affected by concentration. Concentration enhancement will increase the probability of collision between molecules and aggregation, resulting in a decrease in T_cp_. As expected, the T_cp_ was negatively correlated with the concentration of the polymer solution (Figure 4b and Table S1). A slight decrease in T_cp_ was observed following a concentration increase. Further investigation indicated this transition triggered by the environment was totally reversible. An aqueous solution containing (PNAG_26_-b-PNEG_85_)-COOH became cloudy upon heating and became clear again upon cooling. As shown in Figure 4c, a polypeptoid can still revert to its initial phase transition state after eight cycles of heating and cooling between 20 and 70 °C, indicating the good stability of this polymer.

**Figure 5:**
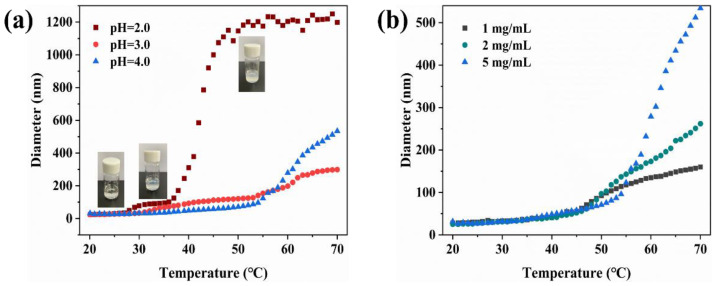
(**a**) Plots of diameter as a function of temperature for aqueous solutions (2 mg/mL) of (PNAG_26_-b-PNEG_85_)-COOH at different pH levels. (**b**) Plots of diameter as a function of temperature for aqueous solutions of (PNAG_26_-b-PNEG_85_)-COOH at pH = 4.0 at different concentrations.

**Figure 6:**
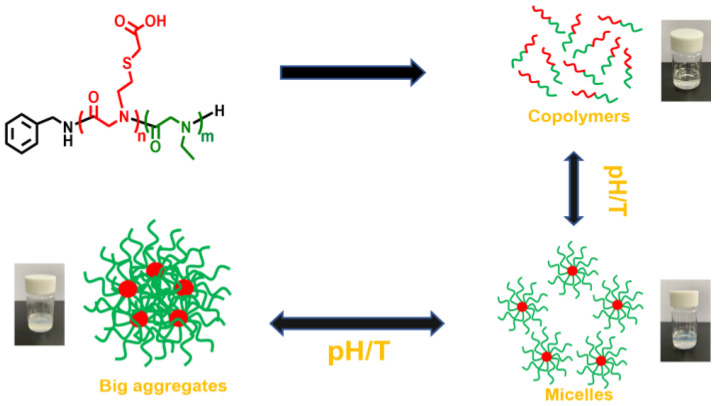
The illustrated process of (PNAG_26_-b-PNEG_85_)-COOH transitioning from free molecules to micelles and big aggregates following pH and temperature changes.

To investigate the self-assembly and aggregation behavior as well as each phase state of the copolypeptoid, DLS was employed to characterize the particle size (hydrodynamic diameter) of (PNAG-b-PNEG)-COOH in solution at various pH values with temperatures ranging from 20 to 70 °C. In Figure 5a, (PNAG_26_-b-PNEG_85_)-COOH assumed different sizes in solutions at varying pH values as well as temperatures. Basically, the particle size increased as temperature rose in the pH range of 2.0–4.0; this finding is consistent with the UV-Vis result revealing that the sample solution underwent a transition from a clear and uniform phase to a turbid aggregation state. Generally, for amphiphilic block copolymers in water, the hydrophobic block aggregates, and the hydrophilic block remains soluble, allowing it to form micelles. Upon increasing hydrophobicity or concentration, individual micelles coalesce into larger aggregates [32]. In this study, the block copolypeptoids experienced a transition from free molecules and micelles to big aggregates in response to temperature and pH change (Figure 5). At pH 2.0, the particle size of the copolymer (PNAG_26_-b-PNEG_85_)-COOH was found to be at the micelle scale (91–125 nm) within the temperature range of 31 to 37 °C. The particle size was small below 31 °C, suggesting free molecules and no micelle formation. Above 37 °C, however, the particle size became increasingly larger, and the polymer formed big aggregates that allowed it to precipitate out from water, resulting in a turbid solution. The reason behind this phenomenon is the solubility change of the PNAG-COOH block. In a copolypeptoid, the PNEG block is hydrophilic and soluble in an aqueous solution for the whole experimental temperature and pH ranges. The PNAG-COOH block, however, is the responsive part, and its solubility is determined by temperature and environmental pH [33]. As shown in Figure 5a, the PNAG_26_-COOH block was hydrophilic when the temperature was low (<31 °C), and the whole block copolypeptoid was soluble in water. As the temperature increased, the PNAG_26_-COOH block became insoluble and aggregated to form a hydrophobic core, while the PNEG_85_ block stayed soluble as a hydrophilic shell. Thus, the block copolymer (PNAG_26_-b-PNEG_85_)-COOH formed micelles in water (31–37 °C). With further temperature enhancement, the hydrophobicity of the PNAG_26_-COOH block increased, causing the micelles to aggregate until precipitation (>37 °C). An illustration of this process is shown in Figure 6. Under different pH conditions (pH = 3.0 and 4.0), (PNAG_26_-b-PNEG_85_)-COOH showed similar phenomena, self-assembling to form micelles at certain temperatures and eventually aggregating into larger particles with the alteration of temperature. It is worth pointing out that a higher temperature was needed to form micelles when the pH value increased, indicating the potential lower hydrophobicity of the PNAG_26_-COOH block in less acidic solutions. In other words, the self-assembly of (PNAG_26_-b-PNEG_85_)-COOH was sensitive to the pH of the environment. The effects of concentration on the self-assembly behavior of the (PNAG_26_-b-PNEG_85_)-COOH samples was also investigated. At high temperatures, the final state of the micellar system was different. In a certain range, a higher concentration was correlated with greater micelle aggregation (Figure 5b). Increasing or decreasing the concentration did not alter the range of internal thermal driving forces necessary for micelle formation [34]. Additionally, polymer structures also affected self-assembly behavior (Figure S4). It was observed that the copolypeptoid with a relatively short PNAG-COOH block showed difficulty responding to the environment. For example, (PNAG_13_-b-PNEG_77_)-COOH and (PNAG_12_-b-NPEG_92_)-COOH did not undergo any changes with a temperature increase at pH 2.0. (PNAG_15_-b-NEG_135_)-COOH, with a long hydrophilic PNEG block, did not show any responsiveness at both pH 2.0 and 4.0. We also noticed that copolypeptoids with relatively short PNAG-COOH blocks can form micelles even at low temperatures. For example, (PNAG_15_-b-NEG_135_)-COOH self-assembled into micelles for the whole experimental temperature range (Figure S4c). This is because a shorter PNAG-COOH block has higher hydrophobicity, and a long PNEG block keeps micelles soluble in solution from further aggregation. These findings agree with the previous report that longer polymer chains with -COOH groups are less hydrophobic. More deionized -COOH groups were embedded in polymer chains and there were fewer hydrophobic residues exposed in aqueous media, which increased the solubility of the polymer [19,29].

The reversible phase transition behavior of the prepared block copolypeptoids in response to temperature and pH indicated excellent stability in solution. In other words, the polymer micelles are stable at a constant temperature and pH. The transition temperature and pH value can be tuned by adjusting the structure of the copolypeptoids to meet different applications. For example, the copolymer will undergo a change in state upon stimulation by temperature or pH, potentially allowing specifically targeted drugs to be loaded and then delivered to organisms. Additionally, drug release from the polypeptoid can occur in response to an organism’s physiological acidity and temperature at the lesion site [35,36,37].

**Figure 7:**
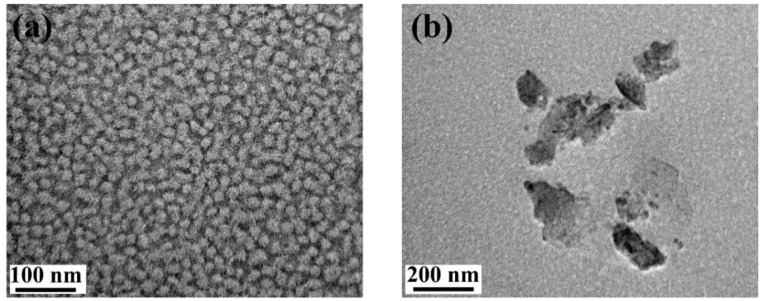
The morphology of (PNAG_26_-b-PENG_85_)-COOH magnified under transmission electron microscope (TEM) at (**a**) 31 °C and (**b**) 50 °C.

In order to determine the micellar structures of the copolymers obtained from the experiments, we observed the microscopic morphologies of the stimulus-responsive diblock copolypeptoid using TEM. (PNAG_26_-b-PNEG_85_)-COOH was disolved in a pH 2.0 solution at temperature of 31 and 50 °C. Then, the solution was dropped and dried on copper grids for a TEM experiment. In Figure 7a, it is obvious that the polypeptoids at 31 °C were presented in round and uniform micelles with a diameter of about 30 nm. The hydrophobic interactions of the PNAG_26_-COOH block drove the formation of micelles at this temperature. As the temperature increased to 50 °C, the micelles clumped together to form larger aggregates measutring over 100 nm, resulting in a more inhomogeneous system (Figure 7b). The TEM result is consistent with the results of the UV-Vis and DLS analysis showing that (PNAG_26_-b-PNEG_85_)-COOH formed micelles at lower temperatures (31 to 37 °C) and big aggregates at higher temperatures (>37 °C). The micelle size difference in the DLS and TEM results can be attributed to the different working principles of these two characterization methods. In DLS, particle size is calculated and generated as hydrodynamic diameter by detecting the light signal scattered by the Brownian motion of particles in solution. In TEM, electrons are used as a light source to diffract a substance, and particle size is measured by taking pictures. In addition, the size obtained from DLS is the hydrodynamic diameter of hydrated micelles in water, but the size from TEM is the diameter of dried micelles. Therefore, both differences in the characterization methods and sample states caused the deviation in the final results.

**Figure 8:**
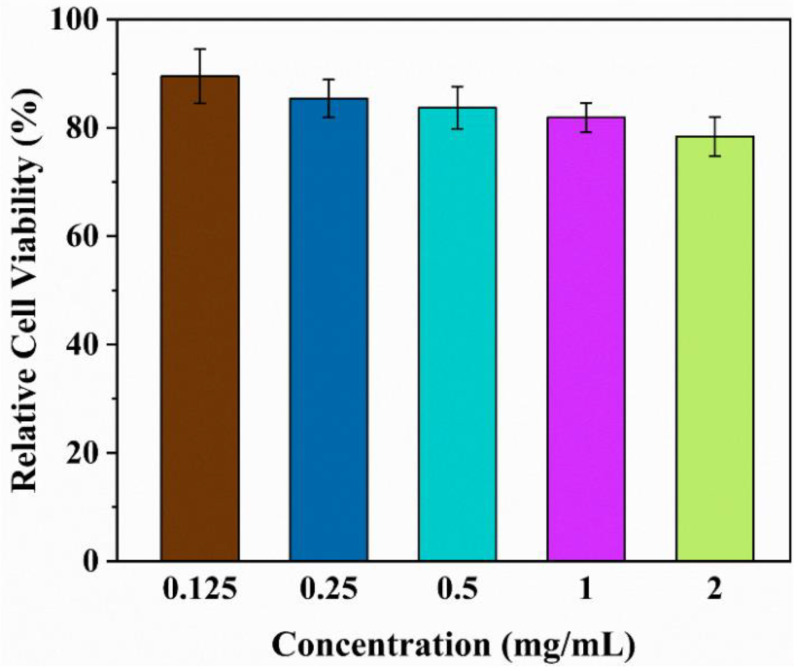
Effect of (PNAG_26_-b-PNEG_85_)-COOH on cell viability at different concentrations.

Polypeptoids have excellent biocompatibility similar to peptides and are expected to have minimal toxicity towards cells. The evaluation of cytotoxicity is a vital benchmark in biological studies. In this study, polypeptoid solutions were prepared at various concentrations (0.125, 0.25, 0.5, 1, and 2 mg/mL) in a complete culture medium (Figure 8). Generally speaking, a sample is considered non-toxic to cells (with no cytotoxicity) when the relative cell viability is above 75% [38]. All the polypeptoid samples prepared in this study demonstrated relative cell viability above 75% for concentrations up to 2 mg/mL, indicating their non-toxicity towards cells. Clearly, our polypeptoid material is non-cytotoxic within the suitable concentration range and has great potential for application in future biomedical material research.

## 4. Conclusions

We developed a series of diblock copolypeptoids with pH and temperature sensitivity by using ring-opening polymerization and click chemistry. The prepared copolypeptoids can self-assemble into micelles reversibly by adjusting the pH and temperature. The self-assembly process during pH and temperature changes was monitored, and all aggregation states as well as mechanisms were proposed. The effects of temperature and pH on the states of copolypeptoid solutions were thoroughly studied. The copolypeptoid micelle formation was also affected by polymer block composition and block length. The self-assembled micelles have excellent stability and can transform back into free molecules by altering temperature and pH values. Due to their favorable biocompatibility and non-cytotoxicity, these block copolypeptoids are expected to be used as biomedical materials for drug encapsulation and delivery trials.

## Figures and Tables

**Table 1:** Synthesis of block copolypeptoids, PNAG-b-PNEG.

Samples ^a^	Feed Ratio ^b^ (Al-NCA:Et-NCA:Initiator)	M_n_^c^ (kg/mol)	M_n_^d^ (kg/mol)	PDI ^d^
PNAG_12_-b-PNEG_92_	15:100:1	8.9	36.7	1.13
PNAG_15_-b-PNEG_135_	15:150:1	12.9	33.6	1.22
PNAG_13_-b-PNEG_77_	15:100:1	7.8	35.9	1.18
PNAG_26_-b-PNEG_85_	30:100:1	9.7	35.3	1.28
PNAG_40_-b-PNEG_80_	50:100:1	10.7	40.1	1.14

^a^ The numbers in subscript are the DPs of each block determined via end-group analysis with ^1^H NMR spectra; ^b^ feed molar ratio of monomers to initiator; ^c^ calculated from ^1^H NMR spectra; ^d^ determined from GPC.

## Data Availability

Data are contained within the article and Supplementary Materials.

## Supplementary Materials

The following supporting information can be downloaded at https://www.mdpi.com/article/10.3390/polym16081082/s1. Scheme S1. Synthetic pathways of Al-NCA and Et-NCA monomers. Figure S1. ^1^H NMR spectra of (a) Al-NCA and (b) Et-NCA in CDCl3 (* indicates solvents). Figure S2. FTIR spectra of Et-NCA, Al-NCA and PNAG26-b-PNEG85. Figure S3. ^1^H NMR spectra of PNAG26-b-PNEG85 in (a) CDCl3 and (b) TFA-d (* indicates solvents). Figure S4. Plots of diameter as a function of temperature for aqueous solutions (2 mg/mL) of (a) (PNAG13-b-PNEG77)-COOH, (b) (PNAG12-b-NPEG92)-COOH and (c) (PNAG15-b-NEG135)-COOH at pH = 2.0 and 4.0. Table S1. The cloud point temperatures of (PNAG26-b-PNEG85)-COOH tested by UV-Vis under different pH values and concentrations.
