# Supplementary material for: Self-Assembly Study of Block Copolypeptoids in Response to pH and Temperature Stimulation
Source: Polymers (Basel). 2024 Apr 12;16(8):1082. doi: 10.3390/polym16081082 (PMC11053516; doi:10.3390/polym16081082)
Supplement: Supplementary file 1 [file polymers-16-01082-s001.zip › polymers-2944426-supplementary.pdf]

Self-Assembly Study of Block Copolypeptoids in Response to pH and Temperature Stimulation

Di Liu, Kang Yang, Liugen Xu, Xiran Shen, Lei Feng, Yangang Jiang, Amjad Ali, Jianwei Lu * and Li Guo *

School of Materials Science & Engineering, Jiangsu University, Zhenjiang 212013, China
* Correspondence: jianwei@ujs.edu.cn (J.L.); liguo@ujs.edu.cn (L.G.)

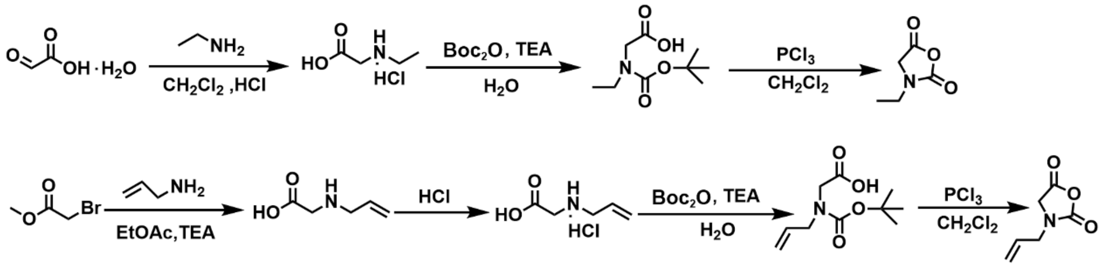

Scheme S1. Synthetic pathways of Al-NCA and Et-NCA monomers.

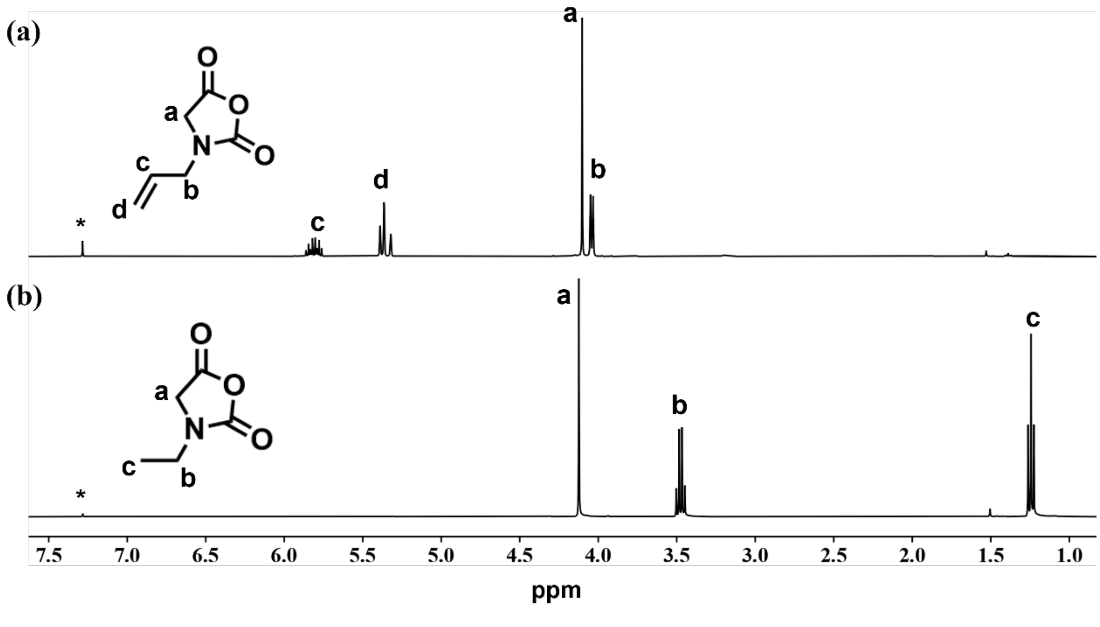

Figure S1. ^{1}H NMR spectra of (a) Al-NCA and (b) Et-NCA in CDCl3 (* indicates solvents).

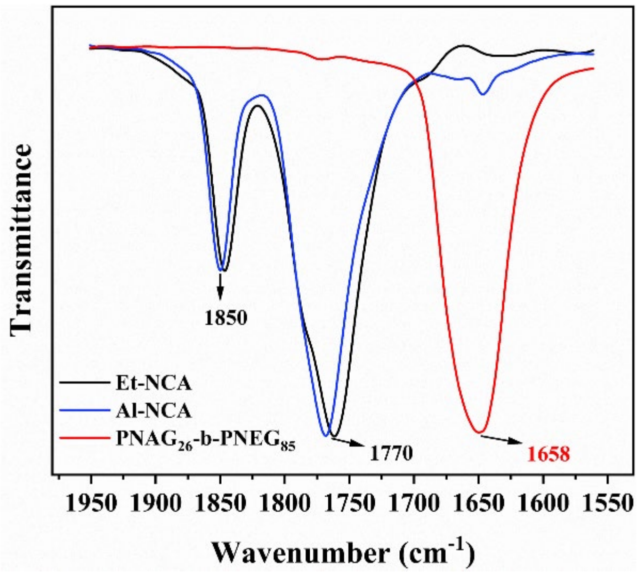

Figure S2. FTIR spectra of Et-NCA, Al-NCA and $PNAG_{26}$-b-$PNEG_{85}$.

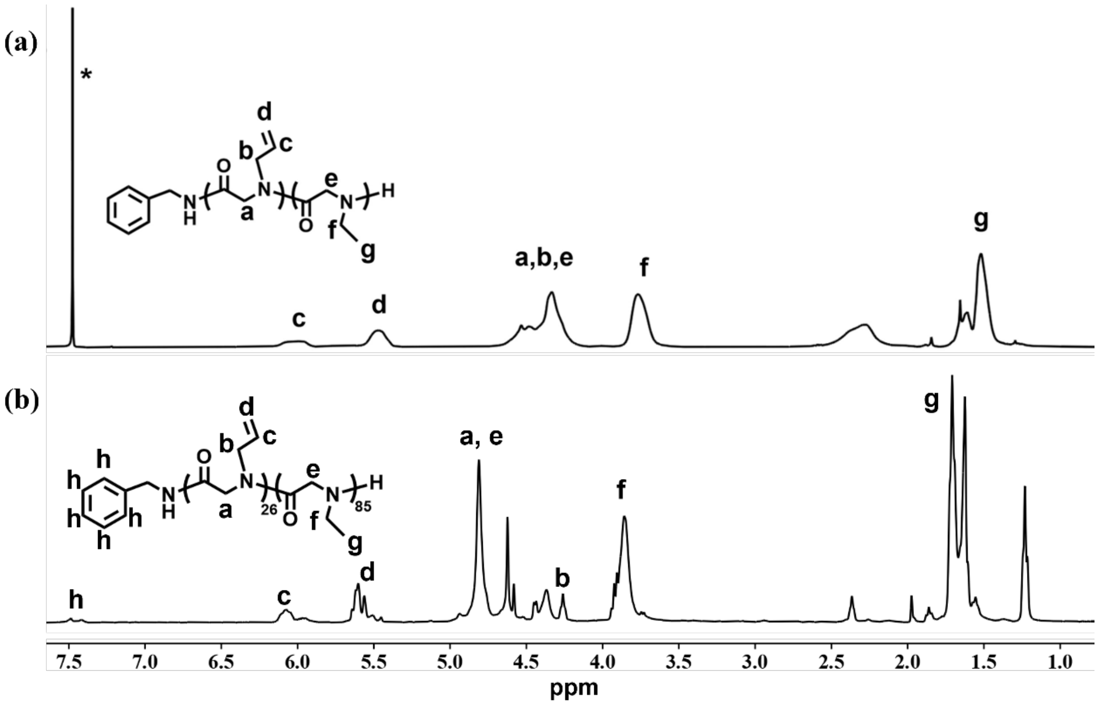

Figure S3. ^{1}H NMR spectra of $PNAG_{26}$-b-$PNEG_{85}$ in (a) $CDCl_3$ and (b) TFA-d (* indicates solvents).

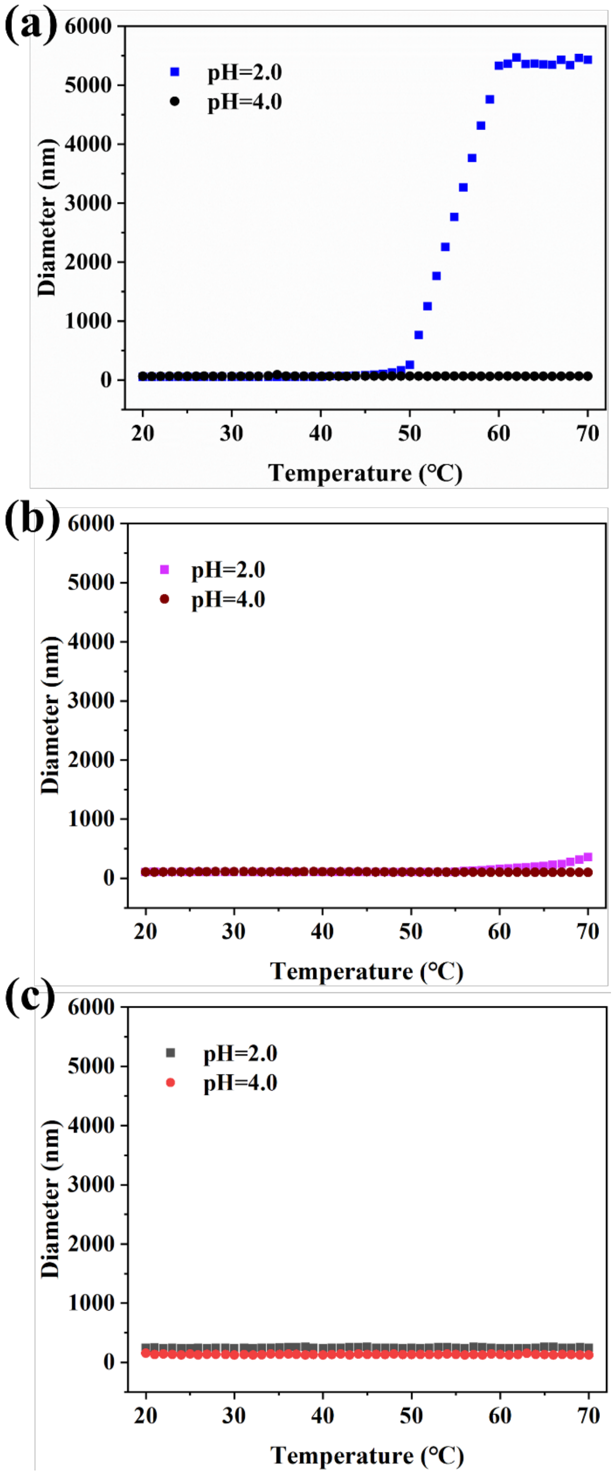

Figure S4 Plots of diameter as a function of temperature for aqueous solutions (2 mg/mL) of (a) ($PNAG_{13}$-b-$PNEG_{77}$)-COOH, (b) ($PNAG_{12}$-b-$NPEG_{92}$)-COOH and (c) ($PNAG_{15}$-b-NEG_{135})-COOH at pH=2.0 and 4.0

Table S1. The cloud point temperatures of ($PNAG_{26}$-b-$PNEG_{85}$)-COOH tested by UV-Vis under different pH values and concentrations

Tcp (°C)	pH	Concentration (mg/mL)
45	4.0	2.0
41	3.0	2.0
37	2.0	2.0
38	2.0	1.0
34	2.0	5.0
